# Elastic Modulus of ECM Hydrogels Derived from Decellularized Tissue Affects Capillary Network Formation in Endothelial Cells

**DOI:** 10.3390/ijms21176304

**Published:** 2020-08-31

**Authors:** Mako Kobayashi, Junpei Kadota, Yoshihide Hashimoto, Toshiya Fujisato, Naoko Nakamura, Tsuyoshi Kimura, Akio Kishida

**Affiliations:** 1 Institute of Biomaterials and Bioengineering, Tokyo Medical and Dental University, Tokyo 101-0062, Japan; mako.mbme@tmd.ac.jp (M.K.); kimurat.fm@gmail.com (J.K.); hashimoto.atrm@tmd.ac.jp (Y.H.); kishida.mbme@tmd.ac.jp (A.K.); 2 Department of Biomedical Engineering, Osaka Institute of Technology, Osaka 535-8585, Japan; toshiya.fujisato@oit.ac.jp; 3 Department of Bioscience and Engineering, Shibaura Institute of Technology, Saitama 337-8570, Japan; naoko@shibaura-it.ac.jp

**Keywords:** d-ECM hydrogels, microvascular-derived endothelial cells, capillary network

## Abstract

Recent applications of decellularized tissue have included the use of hydrogels for injectable materials and three-dimensional (3D) bioprinting bioink for tissue regeneration. Microvascular formation is required for the delivery of oxygen and nutrients to support cell growth and regeneration in tissues and organs. The aim of the present study was to evaluate the formation of capillary networks in decellularized extracellular matrix (d-ECM) hydrogels. The d-ECM hydrogels were obtained from the small intestine submucosa (SIS) and the urinary bladder matrix (UBM) after decellularizing with sodium deoxycholate (SDC) and high hydrostatic pressure (HHP). The SDC d-ECM hydrogel gradually gelated, while the HHP d-ECM hydrogel immediately gelated. All d-ECM hydrogels had low matrix stiffness compared to that of the collagen hydrogel, according to a compression test. D-ECM hydrogels with various elastic moduli were obtained, irrespective of the decellularization method or tissue source. Microvascular-derived endothelial cells were seeded on d-ECM hydrogels. Few cells attached to the SDC d-ECM hydrogel with no network formation, while on the HHP d-ECM hydrogel, a capillary network structure formed between elongated cells. Long, branched networks formed on d-ECM hydrogels with lower matrix stiffness. This suggests that the capillary network structure that forms on d-ECM hydrogels is closely related to the matrix stiffness of the hydrogel.

## 1. Introduction

Decellularized tissue is an extracellular matrix (ECM) assembly comprised of a complex milieu of proteins, polycarbonates, and growth factors that is prepared from living tissue, such as the dermis [1], small intestine submucosa (SIS) [2], urinary bladder matrix (UBM) [3], and the aorta [4]. Decellularized tissue has recently been designated as “decellularized extracellular matrix” (d-ECM). d-ECM is expected to have similar applications as a biomaterial as a natural extracellular matrix. Different types of d-ECMs have been developed using different chemical and physical decellularization methods [5], and surfactants are commonly used. D-ECM is manufactured in sheets or powder, and is typically used for tissue regeneration or repair. Many d-ECM products are already commercially available [6]. Enzymatically dissolved d-ECM forms a hydrogel under certain physicochemical conditions, and could have applications as an injectable [7] or hydrogel patch [8] as a matrix for the support of cellular regeneration, or as a bioink for 3D bioprinting [9]. These versatile d-ECM hydrogels are also used for building three-dimensional (3D) constructs with cells, and as a model for studying cell–matrix interactions.

We have previously developed a novel d-ECM using high hydrostatic pressure (HHP) technology as a physical decellularization method [4,10]. Cells are disrupted by HHP, and the cellular debris is washed out with washing solution containing DNase. Compared to chemically derived d-ECMs, the HHP d-ECM is nontoxic, because no surfactants are used in the decellularization process. We have also identified properties of HHP d-ECM, including tissue structure [10], permeability [11], mechanical characteristics [12], and biocompatibility [13], that are preferable to those of d-ECMs prepared with surfactant methods, such as sodium dodeoxy sulfate (SDS) and sodium deoxycholate (SDC). Furthermore, HHP d-ECM generated using aortic and corneal tissue performed well during in vivo implantation, and resulted in the re-cellularization of recipient cells [4,14]. We propose that HHP d-ECM could be an important biomaterial for tissue repair and regeneration.

Microvascular formation is essential during tissue repair and regeneration for the delivery of oxygen and nutrients to support cell growth and development. The formation of microvasculature has been investigated using hydrogels prepared from synthetic and natural polymers, such as polyethylene glycol [15], polyacrylamide [16], collagen [17], fibrin [18], and gelatin [19]. Hydrogels have been previously engineered specifically to control and enhance microvascular formation. Tabata et al. reported that a gelatin hydrogel, including a growth factor (basic fibroblast growth factor (bFGF)), successfully enhanced microvascular formation because of the controlled release of bFGF from within the hydrogel [19]. Tien et al. developed methods to form and vascularize microfluidic channels within the extracellular matrix [17]. Several groups have reported on the effects of the mechanical properties of the hydrogel on vascular formation. Hammer et al. reported that endothelial cells form stable vascular networks on soft gels [16]. Khademhosseini et al. found that cells cultured on softer regions of hydrogel were round, while cells tended to spread out on stiffer regions of hydrogels [20].

In the present study, we prepared and characterized d-ECM hydrogels using tissue from the UBM and SIS decellularized with SDC and HHP, and investigated the formation of capillary networks in endothelial cells seeded on the d-ECM hydrogels. In particular, we focused on the relationship between the mechanical properties of the hydrogels and the formation of capillary networks, including the length and number of branches of the capillaries.

## 2. Materials and Methods

### 2.1. Materials

Porcine urinary bladder and small intestine were purchased from a local slaughterhouse (Tokyo Shibaura Organ Co. Ltd., Tokyo, Japan) and stored at 4 °C. Phosphate-buffered saline (PBS), sodium deoxycholate (SDC), Dulbecco’s Modified Eagle Medium (DMEM), and DMEM-F12 were purchased from FUJIFILM Wako Pure Chemical Corp (Tokyo, Japan). DNase I, triton X-100, pepsin (≥2500 units/mg), collagenase/dispase, and collagenase type Ⅱ were procured from SIGMA-ALDRICH Japan (Tokyo, Japan). Glutaraldehyde (25%) was obtained from TAAB Laboratories Equipment, Ltd. (Berkshire, United Kingdom). Type I collagen was purchased from Nipponham Foods Ltd. R&D center (Tsukuba, Japan). Calcein-AM solution was purchased from Dojindo Laboratories (Kumamoto, Japan). Alexa Fluor 546 Phalloidin was purchased from Thermo Fisher Scientific, K. K. (Tokyo, Japan).

### 2.2. Preparation of Decellularized UBM and SIS

Porcine urinary bladder tissue was washed in saline solution, and UBM was prepared by mechanically removing the serosal and muscular layers. Porcine small intestine was washed in saline solution and SIS was prepared by mechanically removing the tunica mucosa, tunica serosa, and tunica muscularis externa. The prepared UBM and SIS were sealed in a plastic pack filled with saline solution to prevent implosion and leakage during pressure application. Packs containing tissue were pressurized at 600 and 1000 MPa and 30 °C for 10 min using a hydrostatic pressurization system (Dr. CHEF, Kobe Steel, Ltd., Hyogo, Japan) to dismantle the cells. Propylene glycol was used as the transmission fluid. The pressure-treated UBM and SIS were immersed in saline solution containing DNase I (400 U/mL) and MgCl_2_ (50 mM) at 4 °C for 7 days, followed by washing with 80% ethanol in saline solution at 4 °C for 3 days and in 0.1 M citric acid in saline solution at 4 °C for 3 days.

Surfactant decellularization was performed according to a previously reported procedure [21]. Unless otherwise specified, the treatment temperature and agitation speed were 25 ± 2 °C and 200 rpm, respectively. The UBM and SIS were washed in deionized water at 4 °C for 16 h. Thereafter, the UBM and SIS were washed serially in 0.02% trypsin and 0.05% EDTA at 37 °C for 60 min, 3.0% Triton X-100 for 60 min, 1.0 M sucrose for 15 min, 4.0% deoxycholate for 60 min, and 0.1% peracetic acid in 4.0% ethanol for 120 min. Finally, the UBM and SIS were immersed serially in PBS for 15 min, deionized water for 15 min, deionized water for 15 min, and PBS for 15 min. The decellularized UBM and SIS were stored at 4 °C after lyophilization.

### 2.3. Evaluation of Decellularization and Histological Structure of Decellularized UBM and SIS

The lyophilized UBM and SIS were suspended in a lysis buffer containing 50 µg/mL protease K, 50 mM Tris-HCl, 1% SDS, 10 mM NaCl, and 20 mM EDTA at 55 °C for 12 h. The DNA was extracted with phenol/chloroform and purified by ethanol precipitation. The amount of residual DNA in the native tissue and decellularized tissue were quantified using Quant-iT PicoGreen ds DNA reagent (Thermo Fisher Scientific K. K., Tokyo, Japan) against a λDNA standard curve (0–1000 ng/mL, Thermo Fisher Scientific K. K., Tokyo, Japan) using a microplate reader (excitation 480 nm, emission 525 nm, Cytation 5, BioTek Instruments, Inc., Vinuski, VT, USA).

The HHP and surfactant-decellularized UBM and SIS were fixed with a neutral-buffered (pH 7.4) solution of 10% formalin in PBS at room temperature for 24 h, and then dehydrated gradually in ethanol. Paraffin sections were prepared according to standard procedures. The sections were stained with hematoxylin–eosin (H-E).

### 2.4. Composition of Decellularized Tissue

#### 2.4.1. Collagen Content of Decellularized Tissue

The collagen content of native and decellularized tissue was evaluated using hydroxyproline colorimetric determination. The freeze-dried tissue was transferred to a microtube and then immersed in 6 N HCl and hydrolyzed at 100 °C for 24 h. After hydrolysis, the cap was opened and the tubes were heated at 90 °C to remove the remaining HCl. Each sample was dissolved in 500 µL of assay buffer composed of 5 mg/mL citric acid (monohydrate), 12 mg/mL sodium acetate (trihydrate), 3.4 mg/mL sodium hydroxide, and 1.2 mL glacial acetic acid in distilled deionized water, and subsequently filtered through 0.45 µm PTFE (polytetrafluoroethylene). One-hundred µL of each sample was transferred to a 96-well plate and added to 50 µL of 62 mM chloramine-T solution in 53.3% assay buffer, 26% n-propanol, and 20.7% distilled deionized water. Samples were incubated at room temperature for 15 min and then mixed with 50 µL of Ehrlich’s solution (SIGMA-ALDRICH, Japan), and incubated at 60 °C for 30 min. The amount of hydroxyproline was measured at 550 nm using a microplate reader against a L-hydroxyproline standard curve (0–1000 µg/mL), which was then correlated with the collagen amount using a standard curve and a conversion factor of 7.46 mg collagen to 1 mg 4-hydroxyproline.

#### 2.4.2. Amount of Glycosaminoglycan (GAG) in Decellularized Tissues

The amount of glycosaminoglycan (GAG) in native and decellularized tissue was evaluated by Alcian blue colorimetric assay using an sGAG (sulfated glycosaminoglycan) quantitation kit (EURO DIAGNOSTICA). The tissue was dissolved according to Section 2.3. Aliquots of dissolved tissue (50 μL) were transferred to Eppendorf tubes, resuspended in 50 μL of 8 M guanidine–HCl solution, and incubated at room temperature for 15 min. Samples were mixed with 50 μL of a 0.3% sulfuric acid and 0.75% Triton X-100 solution and incubated at room temperature for 15 min before the addition of 750 μL of Alcian blue solution and incubation at room temperature for 60 min. The supernatant was removed by centrifugation (12,000× *g* at room temperature for 15 min). The resulting pellets were suspended in 500 μL of a DMSO solution (40% dimethyl sulfoxide, 0.05 M MgCl_2_) and incubated at room temperature for 15 min, and then the supernatant was removed by centrifugation (12,000× *g* at room temperature for 15 min). The pellets were dissolved in 500 μL lysis solution (4 M guanidine–HCl, 33% 1-propanol, and 0.25% Triton X-100), and a 240 μL aliquot of each sample was added to a 96-well plate. The amount of sGAG was measured at 620 nm using a microplate reader against a chondroitin 6-sulfate standard curve (0–400 µg/mL).

The lyophilized samples were powdered using a tube mill (IKA Japan K. K., Osaka, Japan). The comminuted samples were then enzymatically digested in a solution of 1 mg/mL pepsin (derived from porcine gastric mucosa, ≥2500 units/mg; SIGMA-ALDRICH, Tokyo, Japan) in 0.01 N HCl under a constant stir rate for 48 h at room temperature, and 10 mg/mL of ECM and d-ECM solutions were prepared. Gelation was induced by neutralizing the pH and salt concentration of the d-ECM solutions at 4 °C, followed by warming to 37 °C. Neutralization of the digestion mixture was accomplished by the addition of 0.1 volumes of 0.1 M NaOH solution, 0.9 volumes of 10× PBS, and dilution to 8 mg/mL ECM with PBS on ice. The neutralized d-ECM solutions were then placed in a non-humidified incubator heated to 37 °C for 1 h to form hydrogels.

### 2.5. Turbidity Measurement during D-ECM Gelation

The gelation kinetics of d-ECM gels were evaluated by turbidity measurement. Neutralized d-ECM solutions at a concentration of 8 mg/mL were prepared on ice. For each sample, 100 µL was added to a 96-well plate, and the absorbance at 405 nm was measured every 3 min for 90 min at 37 °C. The readings were then scaled from 0 (at time 0) to 100% (at maximum absorbance), according to Equation (1), where relative turbidity is the normalized absorbance, *A* is the absorbance at a given time, *A*_0_ is the initial absorbance, and *A_max_* is the maximum absorbance:

(1) $$\mathrm{Relative}\;\mathrm{turbidity}\;=\frac{A-A_{0}}{A_{max}-A_{0}}$$

### 2.6. Scanning Electron Microscopy (SEM) of D-ECM Gels

D-ECM gels at a concentration of 8 mg/mL were fixed in 2.5% glutaraldehyde solution at 4 °C for 24 h. The d-ECM gels were dehydrated gradually with ethanol, immersed in tert-butyl alcohol overnight at room temperature, and dried under reduced pressure. After drying, the d-ECM gels were coated with gold and observed on a scanning electron microscope (S-3400N, Hitachi, Ltd. Tokyo, Japan).

### 2.7. Compression Test

The mechanical properties of the d-ECM gels were evaluated by compression test using a RHEONER Ⅱ (Creep meter RE2-330058, YAMADEN Co., Ltd., Tokyo, Japan). Disks of d-ECM gel (concentration 8 mg/mL, diameter 10 mm, height 10 mm) were prepared. The d-ECM gels were placed on a plate and loaded with a maximum load of 0.2 N and a load speed of 0.1 mm/s.

### 2.8. Primary Rat Brain Microvascular Endothelial Cell (RBMEC) Culture

Animal experiments were approved by the Animal Care and Ethics Committee of Tokyo Medical and Dental University (approval no. A2018273A). Primary rat brain microvascular endothelial cells were harvested and cultured according to a previous report [22]. Briefly, 5-week-old Wistar rats (90–110 g) were purchased from Sankyo Lab Co., Ltd. (Tokyo, Japan) and euthanized by cervical dislocation. The meninges were carefully removed from the forebrains and minced into small pieces of approximately 1 mm^3^ in ice-cold DMEM, dissociated in DMEM containing collagenase type Ⅱ (1 mg/mL) and DNase I (15 µg/mL), and then digested in a shaker at 37 °C for 90 min. The pellet was separated by centrifugation in 20% bovine serum albumin (BSA)-DMEM (1000× *g*, 4 °C, 20 min). The microvascular cells obtained in the pellet were further digested with collagenase/dispase (1 mg/mL) and DNase I (7.6 µg/mL) in DMEM at 37 °C for 45 min. The pellet was added to a 33% Percoll gradient solution (19 mL of 1× PBS, 1 mL of 10× PBS, and 1 mL of fetal bovine serum (FBS)) and washed by centrifugation at 30,000× *g* for 60 min at 4 °C. Microvascular cells were obtained by centrifugation at 1000× *g* for 10 min at 4 °C, and washed twice in DMEM before plating on 35 mm plastic dishes (IWAKI) coated with collagen type IV (0.4 mg/mL) and fibronectin (0.1 mg/mL). Rat brain microvascular endothelial cell (RBMEC cultures were maintained in RBMEC medium I, composed of DMEM/F12 supplemented with 10% FBS, basic fibroblast growth factor (bFGF, Roche, Switzerland, 1.5 ng/mL), heparin (100 µg/mL), insulin (5 µg/mL), transferrin (5 µg/mL), sodium selenite (5 ng/mL) (insulin–transferrin–sodium selenite media supplement, SIGMA-ALDRICH), 1% penicillin/streptomycin, and puromycin (4 µg/mL) at 37 °C in a humidified atmosphere of 5% CO_2_/95% air for 2 days. On the third day, the cells received fresh medium containing all the components of RBMEC medium except puromycin (RBMEC medium Ⅱ). When the cultures reached 80% confluency (on the fourth day in vitro), the purified RBMECs were passaged by briefly treating with 0.05% trypsin–0.02% EDTA solution.

### 2.9. Evaluation of Endothelial Cell Behavior on D-ECM Gels In Vitro

UBM and SIS hydrogels obtained by HHP and surfactant decellularization were prepared at a concentration of 0.8%. A collagen hydrogel was prepared at a concentration of 0.3% as a control. The RBMECs in DMEM/F12 (10% FBS) were seeded on the hydrogels at a cell density of 1.0 × 10^5^ cells/cm^2^. After 1, 3, and 5 days of incubation, the cells were stained with Calcein-AM and Alexa Fluor 546-labeled Phalloidin.

### 2.10. Capillary Network Formation Analysis of RBMECs

The stained RBMECs were analyzed using ImageJ software. The images were binarized, and the length of the network (mm/mm^2^), branch number, and the average width of the network line was calculated using Angiogenesis Analyzer for ImageJ.

### 2.11. Statistical Analysis

Results were expressed as the mean ± standard deviation, with each experiment performed at least three times. Tukey’s multiple-comparison test was used to test for statistical significance. A *p*-value < 0.05 was considered to be statistically significant. For the capillary network formation analysis, the samples were compared for each day.

## 3. Results

### 3.1. Cell Contents After Decellularization

**Figure 1:**
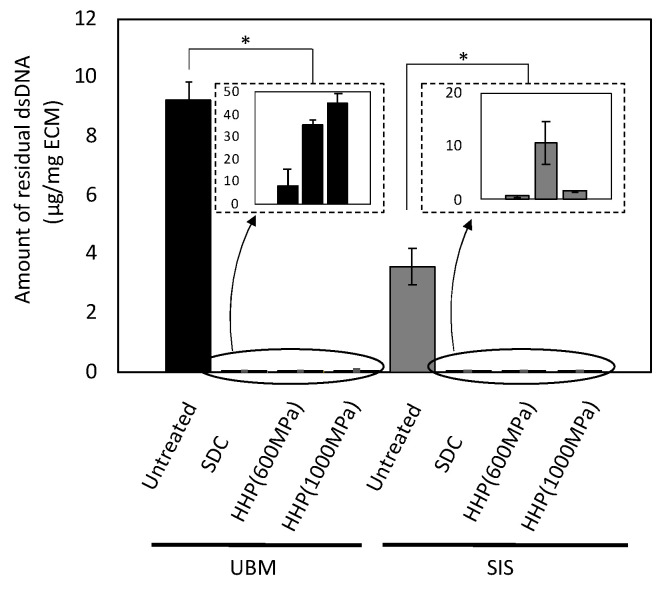
Quantitative analysis of residual double stranded DNA (dsDNA) in decellularized tissue. * *p* < 0.005. SDC and HHP represent the method of decellularization of each tissue (urinary bladder matrix (UBM) and small intestine submucosa (SIS)).

**Figure 2:**
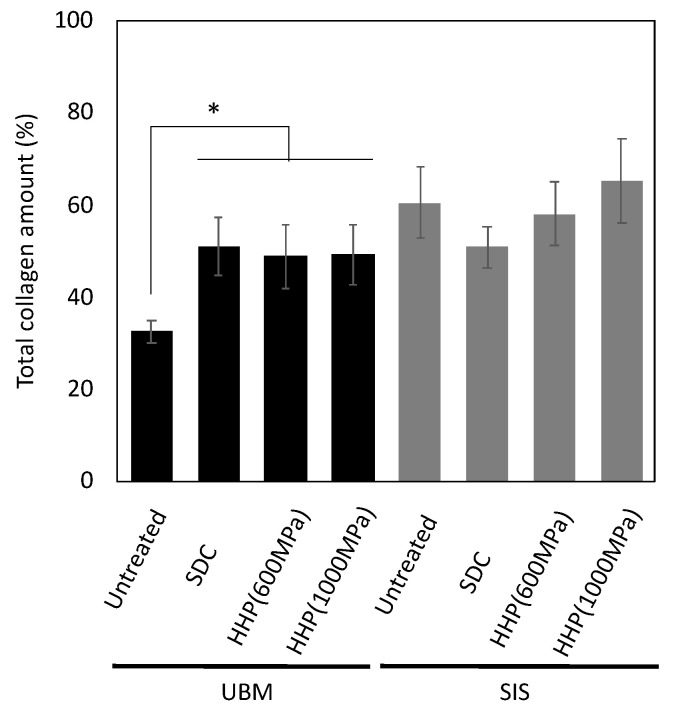
Quantitative analysis of total collagen in decellularized tissue. * *p* < 0.005. SDC and HHP represent the method of decellularization of each tissue (urinary bladder matrix (UBM) and small intestine submucosa (SIS)).

**Figure 3:**
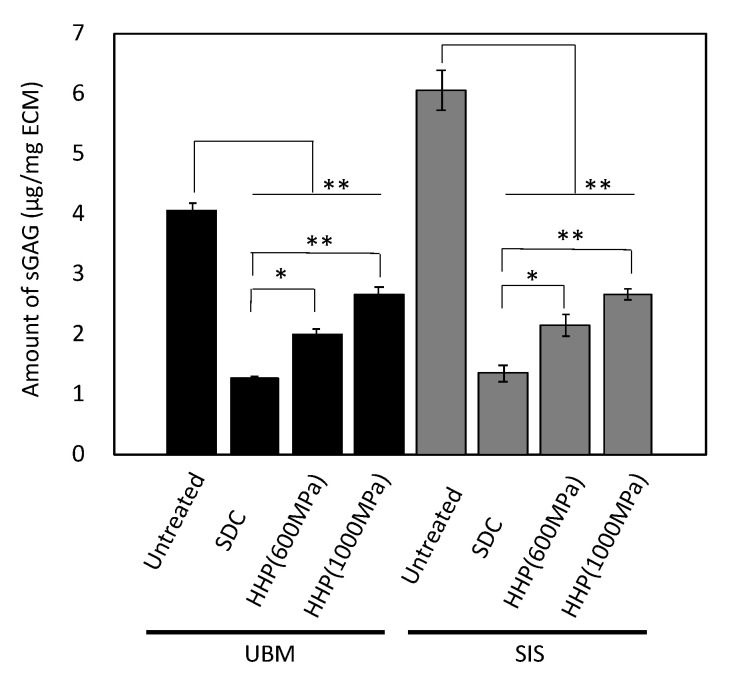
Quantitative analysis of sulfated glycosaminoglycan (sGAG) in decellularized tissue. * *p* < 0.05, ** *p* < 0.01. SDC and HHP represent the method of decellularization of each tissue (urinary bladder matrix (UBM) and small intestine submucosa (SIS)).

UBM and SIS were decellularized with HHP and surfactant, and the remaining DNA was quantified using a PicoGreen assay. The amount of DNA remaining in the decellularized samples was lower than in untreated tissue (Figure 1). The SDC d-ECMs showed a decrease in the amount of residual DNA than the HHP d-ECMs. In the d-ECM from UBM, the collagen content was higher than in the untreated UBM (Figure 2). There was no difference between the collagen content before and after the decellularization of SIS. The amount of sGAG in the d-ECM of UBM and SIS decreased compared to the untreated UBM and SIS for both decellularization methods (Figure 3). The amount of sGAG was similar in the untreated ECM and d-ECM prepared by HHP.

### 3.2. Hydrogel Formation of Decellularized UBM and SIS

**Figure 4:**
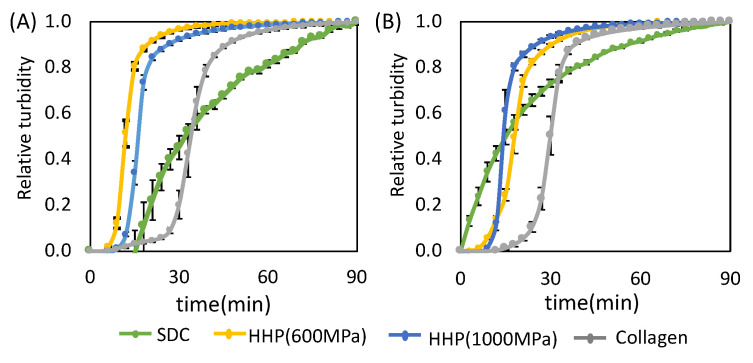
The gelation kinetics of decellularized extracellular matrix (d-ECM) hydrogels and collagen hydrogels. Representative curves are for d-ECM hydrogels derived from (**A**) the urinary bladder matrix (UBM) and (**B**) small intestine submucosa (SIS) at 0.8% concentration. SDC and HHP represent the method of decellularization of each tissue.

Hydrogel formation was evaluated by turbidity measurement. The turbidity curves of the hydrogels of the decellularized UBM and SIS are shown in Figure 4. The rising point of the turbidity curve represents the gel point, and the linear region represents the increase in turbidity associated with gel formation. For the d-ECMs of UBM and SIS prepared by SDC decellularization, the relative turbidity increased gradually until the completion of the experiment. On the other hand, the turbidity of the HHP d-ECMs increased rapidly to a relative turbidity of 0.8, and increased gradually thereafter. The turbidity of the HHP d-ECMs was similar to that of the collagen control.

### 3.3. Characterization of D-ECM Hydrogels

**Figure 5:**
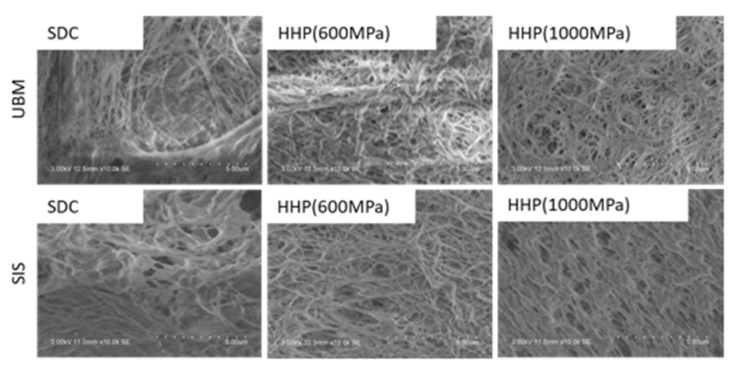
Scanning electron microscopy (SEM) analysis of the surface of d-ECM hydrogels at 0.8%. Scale bar: 5 μm. SDC and HHP represent the method of decellularization of each tissue (urinary bladder matrix (UBM) and small intestine submucosa (SIS)).

SEM was used to visually assess the d-ECM hydrogels obtained from UBM and SIS decellularized with SDC and HHP (Figure 5). Fibers were observed in all d-ECM hydrogels. Few large, heterogeneously formed fibers were observed in the SDC d-ECM hydrogels derived from UBM and SIS. On the other hand, small and uniform fibers were observed throughout the HHP d-ECM hydrogels derived from UBM and SIS. The three-dimensional mesh that formed throughout the HHP d-ECM hydrogel was finer than that of the SDC d-ECM hydrogels derived from UBM and SIS.

**Figure 6:**
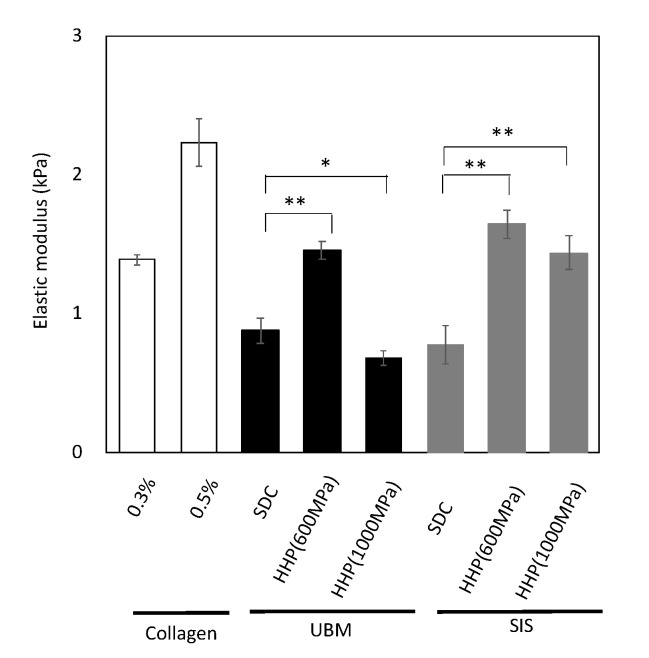
Elastic modulus at 10% strain range of collagen hydrogels and d-ECM hydrogels. * *p* < 0.05, ** *p* < 0.01. SDC and HHP represent the method of decellularization of each tissue (urinary bladder matrix (UBM) and small intestine submucosa (SIS)).

The mechanical properties of the ECM hydrogels were evaluated by a compression test, using 0.8% d-ECM hydrogels with a diameter and height of 10 mm (Figure 6). The calculated collagen concentration in these d-ECM hydrogel pieces was between 0.4% and 0.48%. Hydrogels containing 0.3% and 0.5% collagen (type I) were used as a control. The elastic modulus of the 0.3% collagen (type I) hydrogel was about 1.4 kPa and that of the 0.5% collagen (type I) hydrogel was 2.2 kPa. The elastic modulus of d-ECM hydrogels was between 0.6 and 1.5 kPa, depending on the tissue type and decellularization method. The elastic modulus of all d-ECM hydrogels was lower than that of the collagen hydrogels, although the calculated collagen concentration of the d-ECM hydrogel was higher than that of the 0.3% collagen hydrogel. Among the d-ECM hydrogels prepared in the present study, the SIS-derived sample treated with HHP at 600 MPa had the highest modulus, and the UBM sample treated with HHP at 1000 MPa had the lowest modulus.

### 3.4. Capillary Network Formation of Endothelial Cells on D-ECM Hydrogels

**Figure 7:**
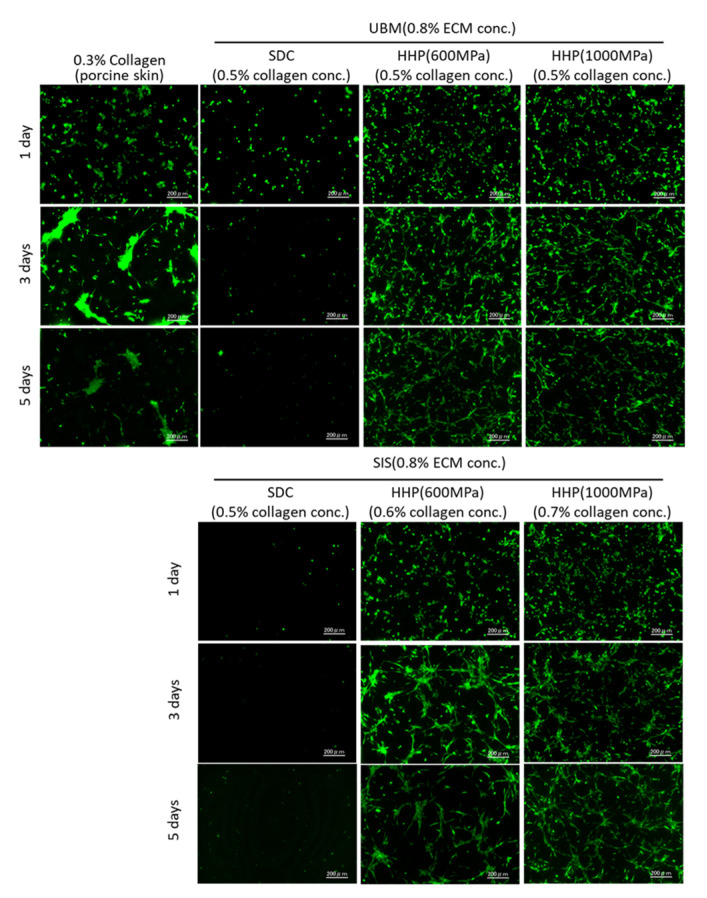
Cell adhesion of rat brain microvascular endothelial cells on 0.3% collagen hydrogels and d-ECM hydrogels at 0.8% d-ECM. The calculated collagen concentration in d-ECM hydrogels is shown in brackets. Green represents live cells stained by Calcein-AM. Scale bar: 200 μm. SDC and HHP represent the method of decellularization of each tissue (urinary bladder matrix (UBM) and small intestine submucosa (SIS)).

Rat brain microvessel-derived endothelial cells were seeded on the d-ECM hydrogels and cultured for 1, 3, and 5 days. The fluorescence of the cells stained with Calcein-AM is shown in Figure 7. For cells on 0.3% collagen hydrogels, cells adhered on day 1 of culture and aggregated on days 3 and 5 of culture. On the SDC d-ECM hydrogels, a few cells adhered on day 1, and most cells disappeared by days 3 and 5 of culture, indicating the low viability of cells on the SDC d-ECM hydrogels. On the other hand, on the HHP d-ECM hydrogels the cells remained viable for 5 days, and appeared elongated and connected without aggregating.

**Figure 8:**
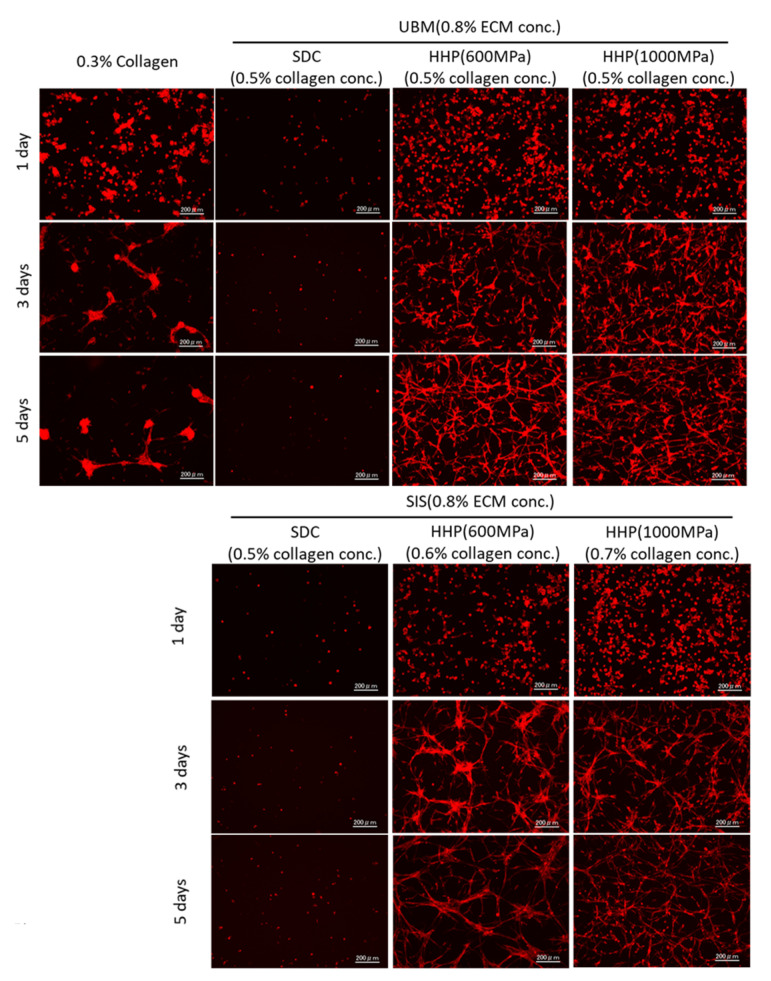
Capillary network structure formed on 0.3% collagen hydrogels and d-ECM hydrogels at 0.8% d-ECM. The calculated collagen concentration in d-ECM hydrogels is shown in brackets. Red represents cytoskeleton stained by Alexa Fluor 546 conjugated phalloidin. Scale bar: 200 μm. SDC and HHP represent the method of decellularization of each tissue (urinary bladder matrix (UBM) and small intestine submucosa (SIS)).

To evaluate the formation of the capillary network by the endothelial cells, F-actin was stained using Phalloidin (Figure 8). On the collagen and HHP d-ECM hydrogels, extended and rounded cells were observed at day 1 of culture. On the collagen hydrogels, cells aggregated and became interlinked at 3 days, with no significant changes after 5 days. On the HHP d-ECM hydrogels, a capillary network structure was formed between elongated cells at 3 days of culture, and the network was still visible at 5 days. The capillary network structure seemed to be dependent on the tissue source. To quantitatively evaluate the formation of capillary networks on the hydrogels, F-actin images were analyzed using angiogenesis analyzer.

**Figure 9:**
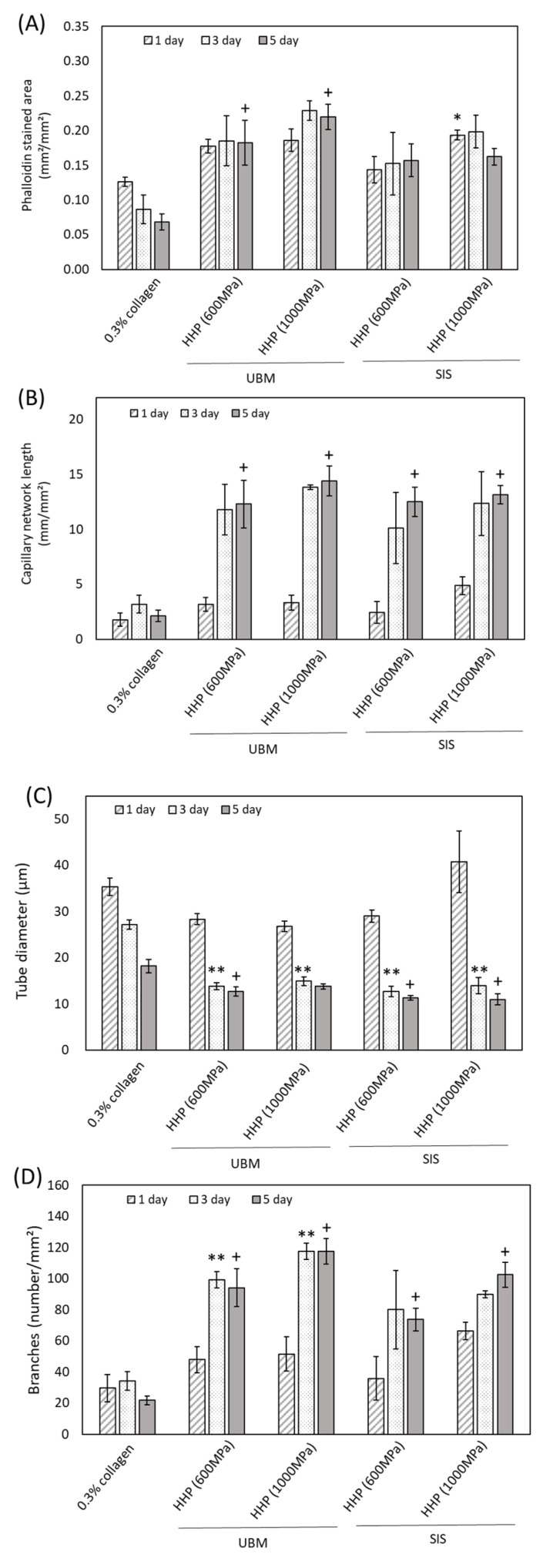
Quantitative analysis of capillary network structure formed on d-ECM hydrogels. (**A**) Phalloidin stained area, (**B**) capillary network length, (**C**) tube diameter, (**D**) branches. The asterisk (*) shows a significant difference (*p* < 0.05) between collagen gel and d-ECM hydrogel at day 1. Two asterisks (**) show a significant difference (*p* < 0.05) between collagen gel and d-ECM hydrogel at day 3. The cross (+) shows a significant difference (*p* < 0.05) between collagen gel and d-ECM hydrogel at day 5. HHP represents the method of decellularization of each tissue (urinary bladder matrix (UBM) and small intestine submucosa (SIS)).

Figure 9A,B show the Phalloidin stained area and the length of each capillary network on 0.3% collagen and HHP d-ECM hydrogels. The area is correlated with the number of cells on the hydrogel. For the collagen hydrogel, the area decreased as the culture time increased because of cell aggregation. On HHP d-ECM hydrogels, a constant capillary network area was maintained for 5 days. The capillary network length increased significantly between days 1 and 3, with no significant changes after 3 days. On SDC d-ECM hydrogels, the area and length were low, irrespective of the number of culture days (data not shown). Figure 9C,D show the diameter of the capillary network and the number of branches, respectively. The diameter decreased over 5 days for all samples. The number of branches were lower in cells grown on collagen hydrogels compared to those grown on HHP d-ECM hydrogels. On HHP d-ECM hydrogels, the number of branches increased significantly from day 1 to day 3 of culture, with no significant changes observed after 3 days. On SDC d-ECM hydrogels, the diameter network and the number of branches was low, irrespective of culture days (data not shown).

**Figure 10:**
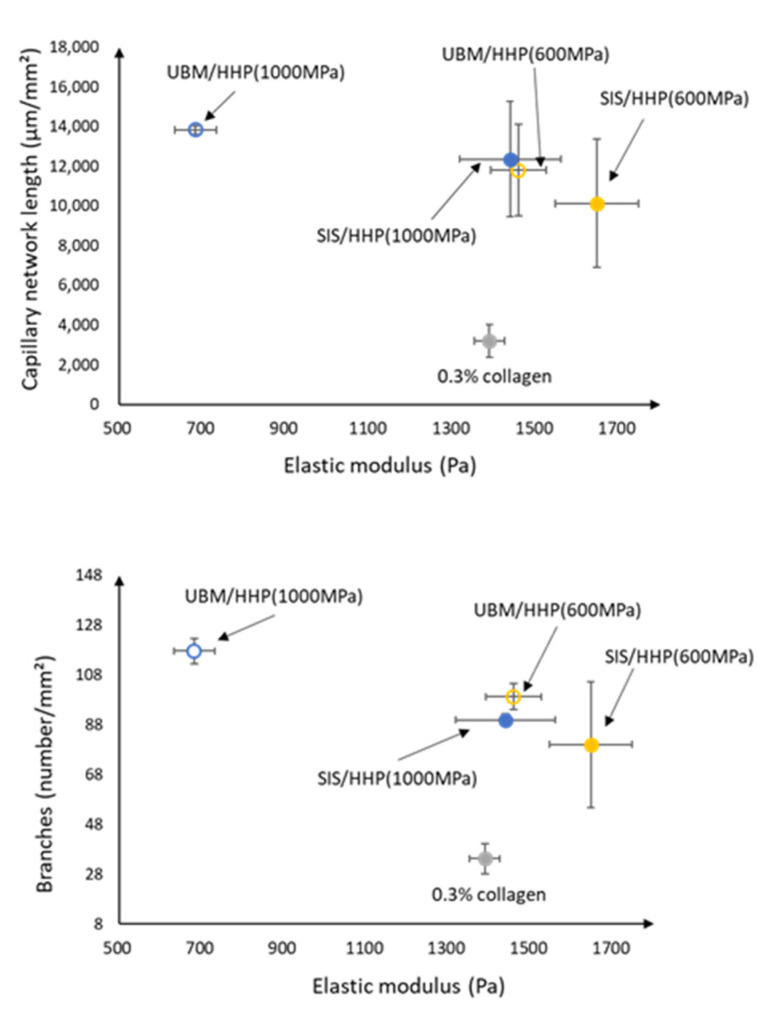
Correlation between matrix stiffness and each parameter of the capillary network structure formed on d-ECM hydrogels. HHP represents the method of decellularization of each tissue (urinary bladder matrix (UBM) and small intestine submucosa (SIS)).

The correlation diagram between matrix stiffness and each parameter of capillary network length and the branch number is shown in Figure 10. On all d-ECM hydrogels, the network length and the branch number of the capillary network structure was higher compared to that of 0.3% collagen. In addition, the capillary network with longer lengths and larger branch numbers tended to form on the d-ECM hydrogel with lower matrix stiffness. On the other hand, the capillary network with short lengths and small branch numbers tended to form on d-ECM hydrogels with high matrix stiffness.

## 4. Discussion

We prepared d-ECM hydrogels using different methods and different tissue types, and characterized their physical properties and the capillary network formation of endothelial cells grown on them. UBM and SIS were decellularized using a traditional SDC method and a detergent-free HHP method, which we have previously published. To understand the effect of hydrogel composition on capillary network formation, we first evaluated the protein composition of decellularized UBM and SIS. In the decellularized SIS, there was no significant change in the amount of total collagen, irrespective of decellularization method, compared to the untreated tissue. On the other hand, in the decellularized UBM, the decellularization treatment resulted in an increase in total collagen content compared to the untreated UBM. The amount of total collagen was determined by measuring the amount of hydroxyproline, which is an amino acid specific to collagen extracted from tissue. The weight of the untreated tissue is made up of both cells and tissue, but the weight of the decellularized tissue is made up of tissue alone. Therefore, the total collagen amount in the decellularized tissue was higher than in the untreated tissue, because the presence of cells reduces the amount of total hydroxyproline by weight. Conversely, both decellularization treatments resulted in a decreased amount of sGAG in the d-ECM samples compared to those in the untreated samples. The amount of sGAG was significantly higher in the HHP d-ECM samples than in the SDC d-ECMs. We also observed lightly stained and fractured fibers in the SDC d-ECM, but not in the HHP d-ECM (Supplementary Figure S1). SDC, which is a strong surfactant, may have dissolved the fibers that remained intact during the gentler HHP treatment. Structural proteins, such as collagen and elastin, are less denatured, and may maintain their native protein conformation in HHP d-ECMs.

d-ECM hydrogels were prepared from decellularized UBM and SIS. The SDC d-ECM hydrogels gradually gelated, while HHP d-ECM hydrogel gelated rapidly. We observed fibers with heterogeneous diameters and three-dimensional structures in the SDC d-ECM hydrogels, while the fibers in the HHP d-ECM hydrogels were relatively homogeneous in diameter and formed a fine three-dimensional mesh. These results suggest that the decellularization treatment strongly affects the formation and structure of d-ECM hydrogels, possibly due to the impact on the protein structure of the constituents. Residual surfactant also inhibits fibrogenesis and fiber formation [23]. SEM images showing heterogeneous fiber structure and cell cytotoxicity for SDC d-ECM hydrogels suggest that surfactant may remain in these hydrogels. We evaluated the mechanical properties of the hydrogels using a compression test, and found that d-ECM hydrogels have a low elastic modulus compared to that of a collagen hydrogel. Among the d-ECM hydrogels analyzed in the present study, the SIS d-ECM hydrogels treated with HHP at 600 MPa showed the highest modulus, and the UBM d-ECM hydrogels treated with HHP at 1000 MPa had the lowest modulus. Although the effect of molecular weight of collagen on elastic modulus was considered, there were no significant differences in the SDS-PAGE band pattern between native collagen and d-ECM hydrogels (data not shown). Previously, several groups have researched the hydrogel formation of collagen mixed with GAG [24,25]. We observed that the elastic modulus of collagen hydrogels did not change when we mixed in GAGs, including hyaluronic acid, chondroitin-6-sulfate, and heparin, but d-ECM hydrogels with added GAGs had a decreased elastic modulus. The differences in mechanical behavior between collagen hydrogels and d-ECM hydrogels is an attractive feature that may have practical applications. In the case of the collagen hydrogel mixed with GAG, the elastic modulus may have remained low because of interactions between collagen and GAGs, while the decrease in the elastic modulus of the d-ECM hydrogels may have occurred because the collagen in the d-ECM had already interacted with GAGs [25]. Further studies, such as the correlation between mechanical properties and porosity, are needed to clarify the detailed mechanisms of d-ECM hydrogel gelation. These results suggest that the method of decellularization for each type of decellularized tissue is important for preparing and controlling the formation of d-ECM hydrogels.

Microvascular-derived endothelial cells were seeded on d-ECM hydrogels prepared from UBM and SIS tissue, and their capillary network structure was evaluated. Few cells attached to the SDC d-ECM hydrogels, and did not proliferate, possibly due to residual SDC in the hydrogels. Insufficient surfactant removal inhibits the repopulation and proliferation of cells. In order to reseed endothelial cells on decellularized tissue, the amount of residual detergents’ concentration needs to be less than 50 μg/mL [26]. On the other hand, many cells adhered to the HHP d-ECM hydrogels and formed a capillary network structure between elongated cells. The network of endothelial cells was observed at 3 days of incubation, and the capillary network was maintained at 5 days of incubation. The network length and the number of branches of the capillary network structure on the HHP d-ECM hydrogels were higher than on the collagen hydrogels. This may be because d-ECM hydrogels contain growth factors, such as VEGF and FGF [27]. Interestingly, the capillary network structure had a longer length and a larger number of branches on the d-ECM hydrogels, even though the matrix stiffness of the d-ECM hydrogels was nearly equal to that of the collagen hydrogels. The lower matrix stiffness of the d-ECM hydrogels resulted in a longer length and larger number of branches in the capillary network. Cell behaviors, such as adherence [28], shape [29,30], and differentiation [31,32,33] are influenced by matrix stiffness. Endothelial cell proliferation and migration is limited on hydrogels with high matrix stiffness, and tube formation capacity is decreased [33]. A similar phenomenon was observed for d-ECM hydrogels in the present study. The capillary network structure on d-ECM hydrogels is strongly related to the matrix stiffness of the hydrogels. These results provide useful insights into capillary vascular formation for tissue engineering using hydrogels formed from decellularized tissue.

## 5. Conclusions

In the present study, we evaluated the capillary network formation of endothelial cells on d-ECM hydrogels by studying their behavior on hydrogels derived from UBM and SIS decellularized using different decellularization methods. Capillary networks with long lengths, many branches, and small diameters were formed on d-ECM hydrogels. The capillary network formation was related to the elastic modulus of the d-ECM hydrogel. These results provide useful insights into microvascular formation for tissue engineering. Further studies into the mechanism of capillary vascular formation on d-ECM hydrogels are needed to enable the control of microvascular formation during wound regeneration and tissue repair.

## Supplementary Materials

Can be found at https://www.mdpi.com/1422-0067/21/17/6304/s1. Figure S1. Photographs of hematoxylin–eosin (H-E) staining of decellularized (A) UBM and (B) SIS with various decellularized methods.

## Abbreviations

ECM	Extracellular matrix
d-ECM	Decellularized extracellular matrix
SIS	Small intestine submucosa
UBM	Urinary bladder matrix
