# Supplementary material for: Elastic Modulus of ECM Hydrogels Derived from Decellularized Tissue Affects Capillary Network Formation in Endothelial Cells
Source: Int J Mol Sci. 2020 Aug 31;21(17):6304. doi: 10.3390/ijms21176304 (PMC7503911; doi:10.3390/ijms21176304)
Supplement: Supplementary file 1 [file ijms-21-06304-s001.pdf]

International Journal of
Molecular Sciences

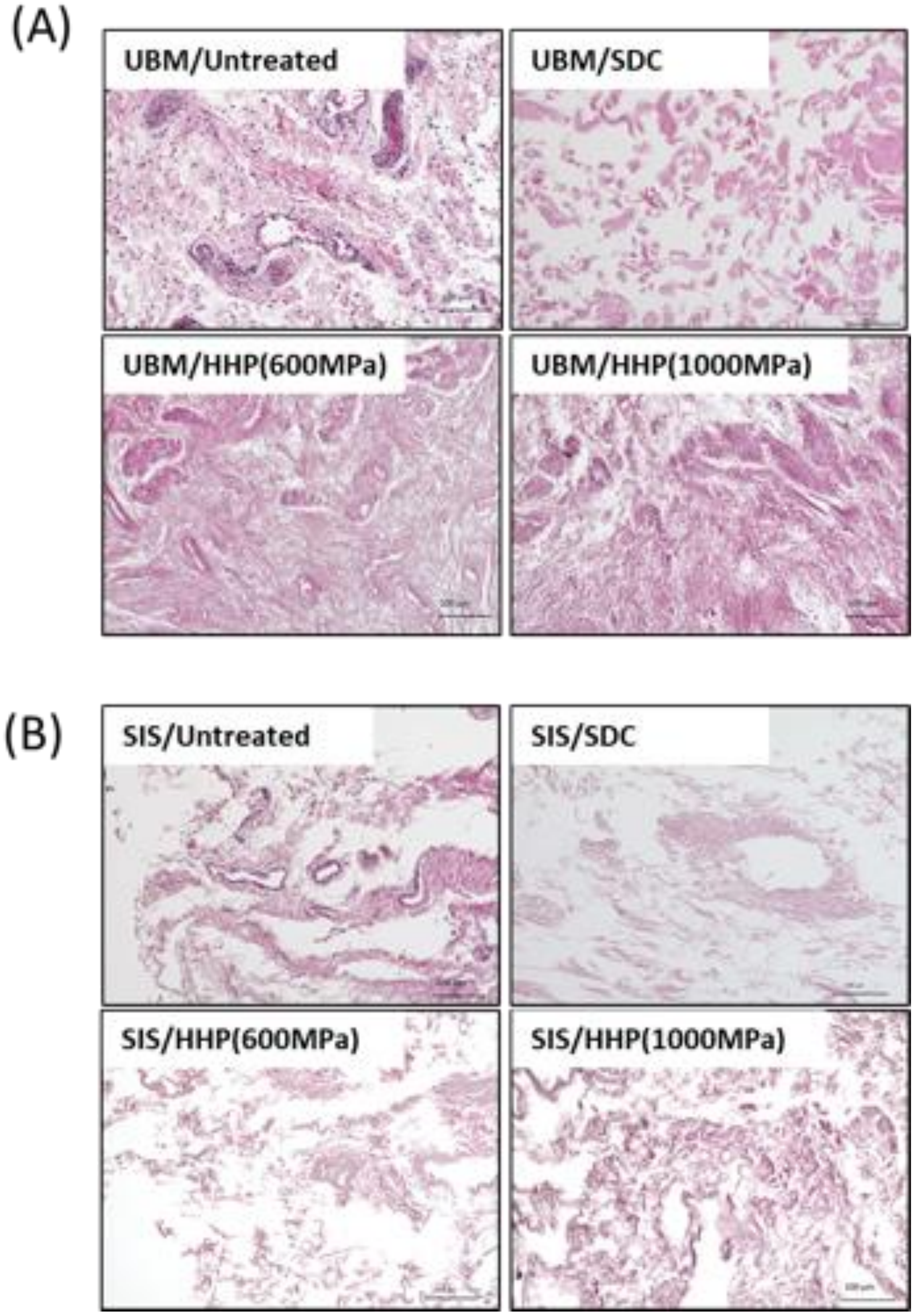

Supplementary Fig 1. Photographs of H-E staining of decellularized (A) UBM and (B) SIS with various decellularized methods.
